# Screening and hit evaluation of a chemical library against blood-stage *Plasmodium falciparum*

**DOI:** 10.1186/1475-2875-13-190

**Published:** 2014-05-27

**Authors:** Vicky M Avery, Sridevi Bashyam, Jeremy N Burrows, Sandra Duffy, George Papadatos, Shyni Puthukkuti, Yuvaraj Sambandan, Shivendra Singh, Thomas Spangenberg, David Waterson, Paul Willis

**Affiliations:** 1 Discovery Biology, Eskitis Institute for Drug Discovery, Griffith University, Nathan, QLD 4111, Australia; 2 Syngene International Ltd, Plot No 2 & 3, Bommasandra IV Phase, Jigani Link Road, Bangalore 560 100, India; 3 Computational Chemical Biology group, European Molecular Biology Laboaratory - European Bioinformatics Institute (EMBL-EBI), European Molecular Biology Laboratory, Wellcome Trust Genome Campus, Hinxton CB10 1SD, UK; 4 Medicines for Malaria Venture MMV, ICC - Block G, 3rd Floor, route de Pré-Bois 20, PO Box 1826, 1215 Geneva 15, Switzerland

**Keywords:** Chemical library, High throughput screening, Phenotypic, Screening cascade, Malaria, *Plasmodium*, Chemical diversity, Hit validation, Cluster, Drug-like, Cheminformatics

## Abstract

**Background:**

In view of the need to continuously feed the pipeline with new anti-malarial agents adapted to differentiated and more stringent target product profiles (e.g., new modes of action, transmission-blocking activity or long-duration chemo-protection), a chemical library consisting of more than 250,000 compounds has been evaluated in a blood-stage *Plasmodium falciparum* growth inhibition assay and further assessed for chemical diversity and novelty.

**Methods:**

The selection cascade used for the triaging of hits from the chemical library started with a robust three-step in vitro assay followed by an *in silico* analysis of the resulting confirmed hits. Upon reaching the predefined requirements for selectivity and potency, the set of hits was subjected to computational analysis to assess chemical properties and diversity. Furthermore, known marketed anti-malarial drugs were co-clustered acting as ‘signposts’ in the chemical space defined by the hits. Then, *in cerebro* evaluation of the chemical structures was performed to identify scaffolds that currently are or have been the focus of anti-malarial medicinal chemistry programmes. Next, prioritization according to relaxed physicochemical parameters took place, along with the search for structural analogues. Ultimately, synthesis of novel chemotypes with desired properties was performed and the resulting compounds were subsequently retested in a *P. falciparum* growth inhibition assay.

**Results:**

This screening campaign led to a 1.25% primary hit rate, which decreased to 0.77% upon confirmatory repeat screening. With the predefined potency (EC_50_ < 1 μM) and selectivity (SI > 10) criteria, 178 compounds progressed to the next steps where chemical diversity, physicochemical properties and novelty assessment were taken into account. This resulted in the selection of 15 distinct chemical series.

**Conclusion:**

A selection cascade was applied to prioritize hits resulting from the screening of a medium-sized chemical library against blood-stage *P. falciparum*. Emphasis was placed on chemical novelty whereby computational clustering, data mining of known anti-malarial chemotypes and the application of relaxed physicochemical filters, were key to the process. This led to the selection of 15 chemical series from which ten confirmed their activity when newly synthesized sample were tested.

## Background

Following the discovery in 1880 of the eukaryote parasite of the genus *Plasmodium* in the red blood cells of malaria patients by the French military doctor, Laveran, malaria remains a widespread tropical disease that affects approximately 207 million people mainly in developing countries. *Plasmodium falciparum, Plasmodium malariae, Plasmodium ovale, Plasmodium vivax* and *Plasmodium knowlesi* are the disease-causing species in humans. Whilst *P. vivax* is responsible for the largest number of malaria infections worldwide, infections by *P. falciparum* account for almost 90% of the burden. With approximately 627,000 deaths per year, it is important to keep in mind that malaria essentially affects infants, children under five years of age and pregnant women, making medicines for paediatric use of major importance [1]. Of continuous concern is the emerging drug-resistance to first-line treatments, such as the endoperoxide artemisinins, stressing the need for a steady pipeline of novel anti-malarial agents [2].

Although much progress has been made in understanding the biology of the parasite lifecycle and mode of transmission, significant challenges still remain, particularly in order to ultimately eradicate malaria, for which new therapeutic agents, as well as transmission and vector control will be needed [3].

With this vision of malaria eradication, several target compound profiles for new medicines can be designed. In addition to being efficacious and safe, all new drugs should ideally be effective against asexual blood stages of malaria. For transmission-blocking purposes, small molecules providing activity against the gametocyte or sexual stages are desirable criteria, as the parasite life cycle will be broken [4]. Importantly, the number of parasites at each stage varies considerably during the replication cycle, suggesting particularly vulnerable points in their lifecycle. Early liver stages in humans and oocytes in the mosquito are two examples of this [5]. Furthermore, activity against the sporozoitic and exo-erythrocytic liver stages of *P. falciparum* and *P. vivax* combined with long duration of action will prevent subsequent re-infection. Finally to attempt malaria eradication, targeting of the dormant hypnozoite liver stage, a relapsing form of malaria caused by *P. vivax*, is essential as this species represents a major health issue outside of Africa. From the perspective of prophylaxis, improving the pharmacokinetic properties of anti-malarial agents will increase protection from re-infection.

In light of these challenges, identifying novel drug-like chemotypes is likely to favour the discovery of compounds with new modes of action (MoA). Herein, validated *P. falciparum* blood-stage active hits for oral drug discovery from a chemical library comprised of 256,263 distinct chemical entities acquired in 2010 are disclosed. In essence, in vitro, *in cerebro* and *in silico* processes involved in the selection and validation of new anti-plasmodial hits are described. The results and analysis is the subject of this article, where the emphasis has been placed on increasing chemical diversity.

## Methods

**Figure 1:**
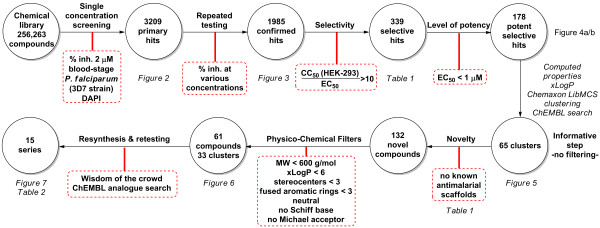
Generation and prioritization of hits from the chemical library.

The following selection cascade was used to triage hits from the chemical library (Figure 1). Notably, chemical novelty and diversity were not evaluated prior to the biological assays. The process started with a robust three-step in vitro assay (spot test, dose response & cytotoxicity) followed by an *in silico* analysis of the resulting confirmed hits. Upon reaching the requirements for selectivity and potency, the set of confirmed hits was subjected to computational analysis to assess chemical properties and chemical diversity. For the latter, commercial anti-malarial agents were included acting as ‘signposts’ in the chemical space defined by the hits. Then, *in cerebro* evaluation of the chemical structures was performed to identify scaffolds that currently are, or have been, the focus of an anti-malarial medicinal chemistry programme. Next, prioritization according to relaxed physicochemical parameters took place, along with the search of structural analogues. The latter were retrieved from the evaluated library, as well as other public domain databases, such as ChEMBL, in order to obtain a better overall picture of the initial structure-activity relationship (SAR) landscape and prior art around the hits [6]. Ultimately, synthesis of novel chemotypes with optimal properties was performed and compounds retested in an independent assay (parasite strain and assay readout being different). In particular, hits featuring so-called bad functional groups (BFGs) were kept until the last stage where they were subjected to a ‘wisdom of the crowd’ approach [7]. Alternatively, removal of the BFG or any other undesirable fragment could be performed during the synthesis step with the risk of activity loss but with a gain in drug-like properties.

### Step 1 ***In vitro*** phenotypic screening

As it has been the case in the majority of recent anti-malarial screening campaigns, the approach used here to identify new hits against *P. falciparum* asexual blood stages utilized phenotypic screening. This approach is advantageous given that currently only a few clinically validated drug targets are available, therefore, increasing the potential for the discovery of new chemotypes acting against new anti-malarial targets [8,9].

The *P. falciparum* 3D7 strain was used for the primary screen. This strain is chloroquine (CQ) sensitive but resistant to the anti-folate drug, sulphadoxine, suggesting a mutation at the dihydropteroate synthase (dhps) level [10]. As a possible consequence, compounds acting on this validated target may not have been identified through this screen.

For the screening of the chemical library, a validated high throughput screening (HTS) method was used as reported by Duffy and Avery [11]. In brief, intra-erythrocyte *P. falciparum* 3D7 parasites incubated with the compounds for 72 hours were stained with 4′-6-diamidino-2-phenylindole (DAPI) and imaged using a high throughput confocal imaging system. To ascertain inhibition of parasite growth, images of fluorescent parasites were translated, using a mathematical algorithm, into a numerical output of classified spots (parasites) as determined by fluorescence size and intensity. The % inhibition of individual compounds was calculated in relation to the activities of the positive and negative controls, 5 μM artemisinin or 0.4% DMSO, respectively. The assay performed with Z’ values generally between 0.5-0.8 and a signal to noise ratio of 10:1 for this screening campaign.

**Figure 2:**
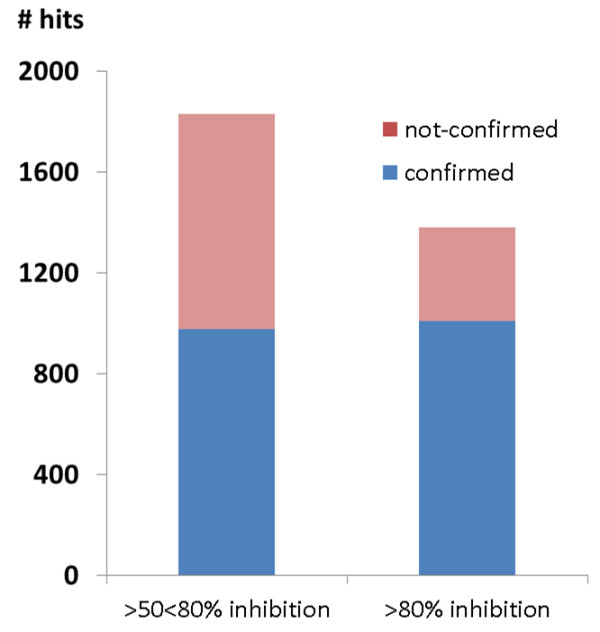
Distribution of confirmed and unconfirmed hits as a function of activity at 1.92 μM.

The library, consisting of 256,263 compounds, was initially tested at 1.92 μM, resulting in the identification of 3,209 primary hits, which exhibited greater than 50% parasite growth inhibition, thus conferring an initial hit rate of 1.25%. A total of 1,829 compounds had inhibitory activities ranging between 50 and 80%, while 1,380 compounds had an activity of greater than 80% at the tested concentration. Upon repeat testing, an attrition rate of 38% was observed refining the set to 1,985 confirmed active compounds. Of note, compounds that had an inhibitory activity between 50 to 80% suffered from a 47% attrition rate, whilst only 28% of compounds that initially displayed an activity greater than 80% did not confirm their activity (Figure 2). This rather high attrition rate for the 50-80% inhibiting compounds is not unusual, considering that many anti-malarial agents display steep EC_50_ Hill slopes. Ultimately, the HTS yielded an overall confirmed hit rate of 0.77%. Primary hits were tested, in a nine-point dose response ranging from 4 to 0.01 μM, against both 3D7 parasites and a human embryonic kidney cell line (HEK293), to determine cytotoxicity (CC_50_) and hence selectivity ratio for the parasite. EC_50_ and CC_50_ values were calculated for compounds which presented an E_max_ plateau using GraphPad Prism. Fifty-eight compounds (17%) did not reach an E_max_ plateau when tested in dose response, and hence accurate EC_50_ values could not be calculated.

**Figure 3:**
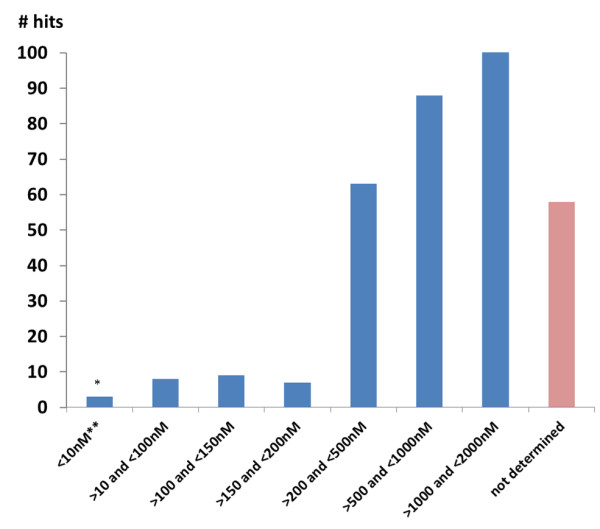
**Number of hits as per activity range against the *Plasmodium falciparum *** **3D7 assay.**

Only compounds displaying at least ten-fold selectivity (CC_50_/EC_50_ ≥ 10) were considered for advancement. The activity range and distribution of compounds demonstrating at least a ten-fold selectivity ratio is presented in Figure 3. Only ten out 339 (3%) of the compounds displayed an EC_50_ below 100 nM. Initially, 52% appeared to have sub-micromolar EC_50_ values whereas 30% of the set proved to have an EC_50_ between 1 and 2 μM.

As detailed by Burrows et al.*,* the early screening of compounds to identify hits for drug discovery programmes suggested that IC_50_ (target-based assay) and EC_50_ (whole-cell assay) values below 1 μM are required to meet the desired hit criteria [3]. By applying this threshold, 178 compounds fulfilled this criterion and displayed no overt toxicity at any dose or had a CC_50_/EC_50_ selectivity ratio greater than ten.

At that stage, not knowing if anti-plasmodial activity would be confirmed or retained by the testing of newly synthesized stocks, prioritization of compounds was not based solely on their HTS potency, rather focus was placed on the quality/structural novelty of each chemotype, along with their physicochemical properties [12].

### Step 2 Overviewing physicochemical properties and assessing chemical diversity

This section describes the efforts to assess and further prioritize hit compounds based on physicochemical, novelty and structural diversity criteria.

For the 178 resulting hits, important physicochemical properties were calculated including molecular weight (MW), partition coefficient (LogP) and topological polar surface area (tPSA). All the calculations and subsequent visualisation and filtering were performed with Dotmatics Vortex software [13].

**Figure 4:**
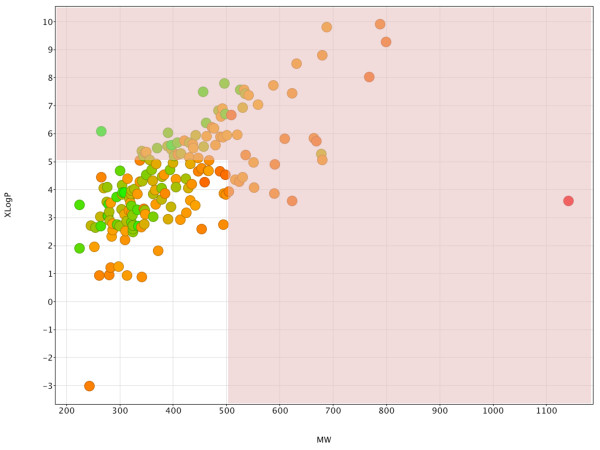
**MW-XLogP scatterplot of the 178 hit compounds, colour-coded by tPSA values.** Green indicates low tPSA and red indicates high tPSA (deep orange indicates tPSA values of around 75A^2^). The red-shaded area denotes the non drug-like space.

**Figure 5:**
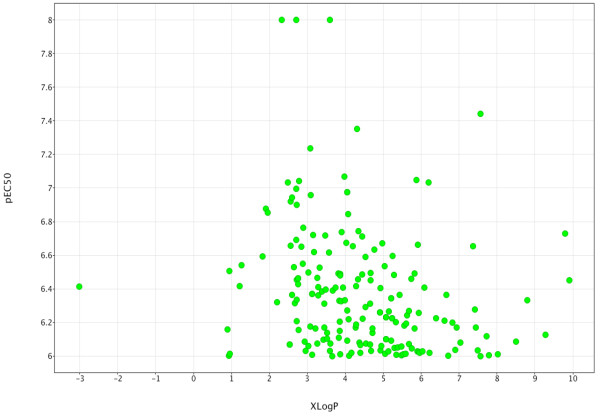
**A scatterplot of pEC**_**50 **_**as a function of XLogP.**

Figure 4 provides an overview of the physicochemical space for the 178 hits. Within the broadly defined drug-like space 107 compounds lie, as they exhibit a MW below 500 g/mol and have a calculated LogP (expressed here as XLogP) below 5 [14]. Plotting pEC_50_ versus calculated XLogP allowed us to see whether lipophilicity was linked to the activity of the set thus avoiding the general trend of high potency with lipophilicity (high XLogP). Figure 5 indicates that high XLogP compounds are not the most active and the majority of the compounds lie within the desired XLogP range of 0 to 5.

**Figure 6:**
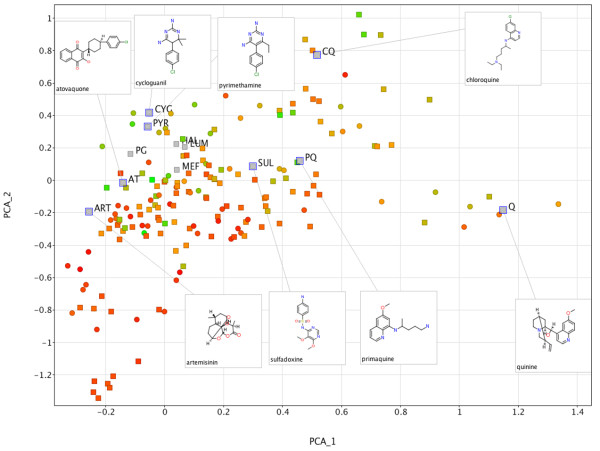
**Two-dimensional Principal Component Analysis similarity scatterplot defining the chemical space of the 178 hits.** Data points represent compounds encoded as 1024-bit Dotmatics FPCA fingerprints on the first two principal component dimensions. The points are colour-coded by LEI, where greener indicates a higher (better) LEI value. For comparison purposes, 12 commercial anti-malarial agents (grey squares) were included in this plot and their respective abbreviation along with their structure is displayed. Points with circular shape indicate compounds that were eventually removed from the next stage.

In order to easily visualize the chemical space defined by the 178 confirmed hits, a principal components analysis (PCA) was performed [15]. Such analysis effectively reduces the dimensions of each data point from thousands to only two or three, thus making visualization in a reduced and interpretable number of dimensions possible. Here, the 178 hits were encoded by a 1024-bit Dotmatics FPCA fingerprint (Figure 6). In addition, 12 commercial anti-malarial agents were included in the plot, acting as ‘signposts’ in the chemical space defined by the hits. The latter are represented by grey squares, whereas data points with a circular shape indicate compounds that were eventually removed at the next stage. This aided the selection of chemotypes lying in different areas of chemical space than those which are currently used as anti-malarials. To further guide the selection an indicative Ligand Efficiency Index (LEI) [16,17] has also been added to the display (vide infra).

Also, with regard to the physicochemical properties calculation, structural clustering was performed in order to minimize structural redundancy and identify a subset of chemically diverse hits. The analysis was performed using LibMCS clustering provided by Chemaxon [18] and implemented in a Pipeline Pilot (Accelrys®) protocol. This method clusters compounds by attempting to maximize the size of the shared substructure among them and thus tends to generate results that are closer to chemical intuition. As expected, LibMCS clustering yielded 65 clusters sharing the same scaffold/chemotype, each featuring one to 12 compounds.

### Step 3 Removing known anti-malarial chemotypes

Active hits were subjected to a substructure search to identify known chemotypes occurring in approved anti-malarial therapeutics, new chemical entities under development and other published anti-malarial drug discovery efforts. As a result, 40 out of the 178 compounds were flagged (22%) and subsequently deprioritized. However, the presence of these known actives validates both the assay, and the selection and clustering procedures, and increases belief in the potential value of the novel chemical matter identified.

Some of the scaffolds used in the substructure searching, along with their frequency in the hit list, are depicted in Table 1 (NB: Not all the scaffolds are displayed for confidentiality reasons). The common anti-malarial quinoline fragment [19] was found in the hit list indicating the presence of close analogues of quinine, mefloquine, chloroquine, quinacrine, and amodiaquine. Furthermore, a significant number of analogues relating to published anti-malarial chemotypes, such as diamino-pyrimidines [20-22], triazolo-pyrimidine [23,24] or purines [25], were identified.

**Table 1:** Examples of substructures and scaffolds of known anti-malarial agents and their frequency of occurrence in the hit list

**Scaffolds**		**Counts**	**Comments**
A		9	Quinine or mefloquine like
B		9	Diamino-pyrimidines
C		4	Pyrimethamine or cycloguanil like
D		4	Triazolo-pyrimidine
E		3	Purine
F		2	Chloroquine, quinacrine or modiaquine like
G		1	Chloroproguanil or proguanil like

### Step 4 Filtering hits with undesirable physicochemical properties and functional groups

To avoid general developmental, promiscuity and toxicity issues associated with high molecular weight and lipophilicity, [26] compounds with molecular weights above 600 g/mol or XLogP above 6 were removed.

**Figure 7:**
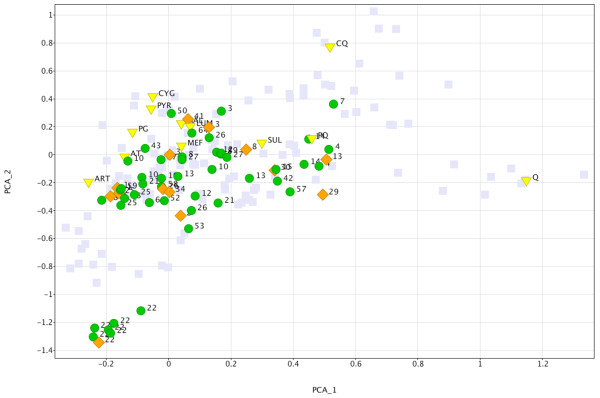
**Two-dimensional Principal Component Analysis similarity scatterplot defining the chemical space of the 178 initial hits.** Details as per 61 remaining compounds by cluster number. Each point is a compound encoded as 1024-bit Dotmatics FPCA fingerprint. Yellow triangles denote commercial anti-malarial drugs along with their abbreviation. Green circles denote hits that passed all the physicochemical, novelty and substructural filters, while the orange rhombi denote compounds that belong to the final validated hit set. The subset of the initial 178 remaining compounds that did not advance through the cascade is highlighted in light grey.

Moreover, after further examination of the hit list, compounds with undesirable features, including those with more than two stereogenic centres, more than two fused aromatics rings, Michael acceptors or Schiff bases, were manually flagged and eliminated, leaving 61 compounds for consideration (Figure 7). The latter were successively submitted as queries for similarity searching in ChEMBL using a KNIME workflow [27]. Overall, 36 out of the 61 hits had one to 219 distinct near neighbours within a 0.85 Tanimoto similarity threshold (using the standard MDL Symyx database fingerprints provided by the ChEMBL interface), while 15 were exact matches, already reported in ChEMBL. This novelty assessment was taken into consideration during the ‘wisdom of the crowd selection’, where MMV’s experience in medicinal chemistry and knowledge of the current project portfolio was explicitly factored in.

### Step 5 Synthesizing and retesting of selected chemotypes

As chemical stability and structural confirmation can never be guaranteed, reconciliation of the chemical and biological characteristics of a compound is essential for hit validation. Here chemical synthesis and further in vitro testing was used to validate the hits. For details about specific methodologies used, see Additional file 1.

Synthesized compounds were tested in an alternative assay, in which ^3^H-hypoxanthine incorporation was measured in the chloroquine-sensitive strain NF54 (Values were measured in duplicate, 12 points/EC_50_). Since different strains, as well as different readouts, were used, direct comparison of EC_50_ values cannot be performed; however, this independent and orthogonal retention of overall activity strongly corroborates the initial hit.

Although synthesis of hit compounds is time-consuming, it provides the first insights into the synthetic complexity and is essential to ascertain that the activity reported is associated with the specific compound. In addition, this established the essential platform for subsequent rapid synthesis of new members aimed at the removal of potential undesirable functional groups (with possible toxicity liabilities) and or the substitution of more suitable groups to optimize the physicochemical properties (e.g., LogP). Whilst modifications may result in some loss of activity, the benefit would be a compound with more drug-like physicochemical attributes that otherwise would have been deprioritized or discarded.

**Figure 8:**
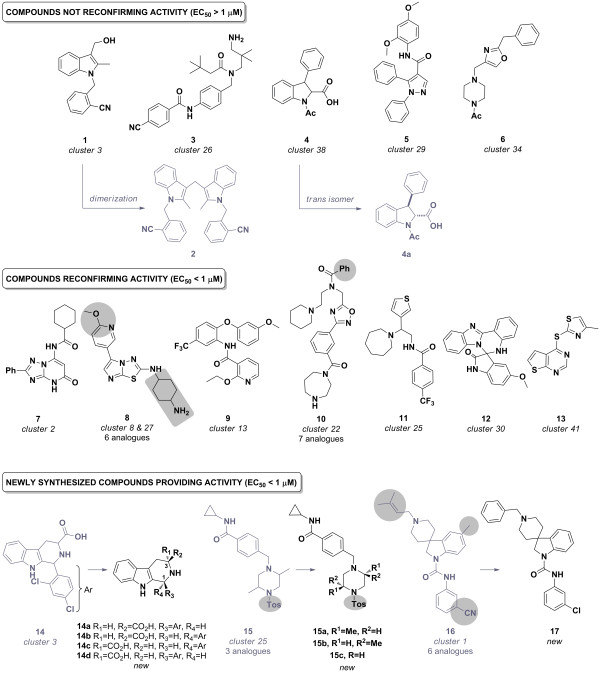
**Selected clusters and compounds for synthesis.** Grey areas indicate structural variation among the analogues present in the hit set.

Figure 8 shows the lead chemical scaffold selected for each remaining cluster. Importantly, areas in grey represent structural changes in the same series. Table 2 summarizes essential physicochemical data along with in vitro biological results.

**Table 2:** Summary of compounds with their identification number, main physicochemical properties and bioactivities

**Compound**	**MW (g/mol)**	**xLogP**^**a)**^	**tPSA NOPS**^**a)**^	**EC**_**50**_**in nM**^**b)**^**(3D7)**	**EC**_**50**_**in nM**^**c)**^**(NF54)**	**pEC**_**50**_^**d)**^**(3D7)**	**pEC**_**50**_^**d)**^**(NF54)**	**LEI**^**e)**^**(3D7)**	**LEI**^**e)**^**(NF54)**	**Comment**
**1**	276.53	4.1	49	602	4700	6.2	5.3	22.5	19.2	Weakly active/unstable
**2**	504.62	9.5	57	ND	763	N/A	6.1	N/A	12.1	-
**3**	434.57	4.2	99	222	3000	6.7	5.5	15.3	12.7	Weakly active
**4**	281.81	2.9	58	172	ND	6.8	N/A	24.0	N/A	-
**4a**	281.81	2.9	58	ND	>10000	N/A	<5	N/A	<17.7	Inactive
**5**	399.44	5.0	65	870	3898	6.1	5.4	15.2	13.5	Weakly active
**6**	299.37	2.7	50	<10	>10000	>8.0	<5.0	>26.7	<16.7	Inactive
**7**	337.38	4.3	89	648	450	6.2	6.3	18.3	18.8	Validated hit, CHEMBL587310^f)^
**8**	344.43	3.3	119	297	284	6.5	6.5	19.0	19.0	Validated hit
**9**	432.39	4.9	70	393	309	6.4	6.5	14.8	15.1	Validated hit
**10**	516.28	4.4	96	181	60	6.7	7.2	13.0	14.0	Validated hit
**11**	396.47	5.4	61	433	518	6.4	6.3	16.1	15.9	Validated hit
**12**	368.39	4.9	68	928	60	6.0	7.2	16.4	19.6	Validated hit, CHEMBL1545915^f)^
**13**	265.38	4.4	120	194	102	6.7	7.0	25.3	26.3	Validated hit
**14**	361.22	4.3	65	350	ND	6.5	N/A	18.0	N/A	CHEMBL527593^f)^
**14a**	361.22	4.3	65	ND	>10000	N/A	<5	N/A	<13.8	Inactive
**14b**	361.22	4.3	65	ND	167	N/A	6.8	N/A	19.7	Hit
**14c**	361.22	4.3	65	ND	4200	N/A	5.4	N/A	14.9	Weakly active
**14d**	361.22	4.3	65	ND	>10000	N/A	<5	N/A	<13.8	Inactive
**15**	441.59	3.4	78	678	ND	6.7	N/A	14.0	N/A	-
**15a**	441.59	3.4	78	ND	44	N/A	7.4	N/A	16.7	Hit
**15b**	441.59	3.4	78	ND	679	N/A	6.2	N/A	14.0	Hit
**15c**	413.53	2.4	78	ND	535	N/A	6.3	N/A	15.2	Hit
**16**	414.54	5.3	59	990	ND	6.0	ND	12.1	N/A	-
**17**	431.96	6.2	36	ND	309	ND	6.5	N/A	15.1	Hit

ND: not determined; N/A: not applicable; ^a)^calculated with the Dotmatics Vortex software; ^b)^EC_50_ determined with the *P. falciparum* 3D7 strain with DAPI imaging (HTS screen) *see experimental*; ^c)^EC_50_ determined with the *P. falciparum* NF54 strain by ^3^Hypoxanthine incorporation (Hit validation) *see experimental*; ^d)^half maximal effective concentration (EC_50_) expressed as a negative logarithm of the molar concentration; ^e)^Ligand Efficiency Index (LEI) = pEC_50_/(MW/1000); ^f)^CHEMBL_ID upon successful search in ChEMBL Malaria. NB: **12** (MMV396749) and **14** (MMV008138) were placed in the public domain in July 2011.

## Results

### Compounds whose activity could not be reconfirmed (EC_50_ > 1 μM)

**1** (cluster 3) is an indole derivative which showed moderate activity with an EC_50_ of 602 nM during the HTS campaign, and was an attractive hit molecule based on low molecular weight and, therefore, a high Ligand Efficiency Index (LEI). Upon synthesis, this compound showed a significant reduction in activity as the EC_50_ was only 4.7 μM against the *P. falciparum* NF54 strain. Notably, the compound significantly degraded upon standing at room temperature to the dimer **2** (Figure 8). Once retested, this compound showed an EC_50_ in the same range as initially described during the HTS, suggesting that the original compound sample had degraded over time. Due to this obvious chemical instability, indole derivative **1** did not qualify as a hit. **3**, (cluster 26) is a structurally simple molecule, comprised of two amide bonds and a primary amine group, which post synthesis demonstrated a significant decrease in activity from an initial EC_50_ of 222 nM (*P. falciparum* 3D7) to 3 μM (*P. falciparum* NF54). **4** (Cluster 38) has an indoline core with two adjacent stereocentres and was originally tested at HTS as a potential mixture of four diastereomers. The racemic mixture of the thermodynamically more stable *trans* compounds **4a** was therefore synthesized using a known route [28]. Unfortunately both enantiomers were inactive in the confirmatory assay. Activity of the *cis*-diasteromers cannot be excluded but its synthesis posed a significant chemical challenge. **5** (cluster 29), a substituted 1*H*-pyrazole, with an initial EC_50_ of 870 nM demonstrated reduced activity after synthesis with an EC_50_ of almost 4 μM. This compound therefore did not qualify as a hit according to the predefined criteria. **6** (Cluster 34), 1-(4-((2-benzyloxazol-4-yl)methyl)piperazin-1-yl)ethanone, was found to be the most potent compound identified during the HTS campaign with an EC_50_ value of <10 nM along with desirable physicochemical properties. Unfortunately, upon synthesis this compound was shown to be inactive (NF54 EC_50_ > 10 μM) suggesting that this compound was potentially a false positive.

### Compounds with reconfirming activity (EC_50_ < 1 μM)

**7** (Cluster 2), a 7-amino- [1,2,4] triazolo [1,5-a]pyrimidin-5 (4*H*)-one derivative, showed consistent anti-malarial activity both at HTS and after synthesis with EC_50_ values ranging from 450 to 648 nM, and therefore provided a good chemical starting point. **8** (cluster 8, analogues also found in cluster 27) is comprised of a imidazo-[2,1-b]-[1,3,4]-thiadiazole core and has six analogues present in the set of 178 compounds. Upon retesting, this compound confirmed activity, 284–287 nM, with good physicochemical properties and a high LEI of 19. **9** (cluster 13), a *N*-phenylnicotinamide derivative, showed consistent activity between 309 and 393 nM and is amenable to chemical modifications. **10** (cluster 22) comprised of 1,2,4-oxadiazole core, is a novel but relatively large molecule that confirmed activity with EC_50_ values ranging from 60 to 181 nM. Although racemic, **11** (cluster 25), embedded with an 1-(thiophen-3-ylmethyl)-azepane moiety, has displayed a similar degree of activity with EC_50_ values of 433 nM and 518 nM in the two independent assays. With a potential to increase the potency of one of the isoforms, along with having a small molecular weight, this compound looks promising as a starting point for lead optimization. **12** (cluster 30) is a racemate with a spiro group along with a 2-phenyl-1*H*-benzo-[d]-imidazole moiety. The molecule showed sub micromolar potency in the HTS assay (928 nM) and showed a significant increase in potency upon synthesis and independent retesting with an EC_50_ of 60 nM (*P. falciparum* NF54 strain) demonstrating an LEI of 19.6 and, therefore, an excellent starting point for a medicinal chemistry programme. Although it belongs to a different chemical class, **12** bears a significant structural similarity to KAE609, a spiroindolone currently in phase 2 for the treatment of malaria [29,30]. **13** (cluster 41) a sulphur atom containing compound with a thieno-[2,3-d]-pyrimidine core, is a small molecule which showed a constant high potency in both assays with an EC_50_ ranging from 102–194 nM. Due to its low molecular weight, this compound is an ideal starting point, having an LEI of 26.3.

### Newly synthesized compounds with activity (EC_50_ < 1 μM)

As previously stated, **14** is likely to be a mixture of four diastereomers of a β-tetrahydrocarboline derivative. Firstly, the synthesis and testing of the corresponding natural amino acid, L-tryptophan, was performed, then subsequently the major and thermodynamically more stable *trans* isomer **14b**[31]. The in vitro EC_50_ against *P. falciparum* NF54 was 167 nM as compared to 350 nM for the mixture (**14**) and displayed an LEI of 19.7. Of note is that the other three isomers were subsequently isolated/synthesized and tested. **14a** and **14d** were inactive and **14c**, the *cis* isomer from D-tryptophan, only demonstrated weak activity, EC_50_ = 4.2 μM. **15**, a 2,5-dimethylpiperazine sulphonamide, showed moderate activity at HTS with an EC_50_ of 678 nM and had two unassigned stereocentres on the heterocycle. Synthesis with the readily available *trans* 2,5-dimethylpiperazine was performed, leading to the racemate which was subsequently separated on a chiral HPLC column to yield **15a** (2*R*,5*S*) and **15b** (2*S*,5*R*). Interestingly, the two enantiomers showed a difference of more than 15-fold in activity, with EC_50_ value of 44 nM and 679 nM, respectively. Also removal of the two methyl groups on the piperazine ring of **15** helped to simplify the structure and reduce lipophilicity. This led to **15c**, which exhibited an EC_50_ of 535 nM and which is still an attractive starting point for a medicinal chemistry programme due to its novelty and structural simplicity. Racemic **16** (cluster 1) contains an attractive spiro [indoline-3,4′-piperidine] fragment with a potency just below the 1 μM threshold. Six analogues were present in the set of 178 hit compounds. Of particular note, using strict ‘rule of five’ filters during the selection process would not have enabled the retrieval of this chemotype. Relying on the structures of the analogues present in the hit set during resynthesis, the cyano group was replaced by a chlorine atom as well as the substituted allyl group by a benzyl group. Finally, in an attempt to simplify the core structure the methyl group on the indoline moiety was removed to yield **17**, a compound that maintained activity with an EC_50_ of 309 nM as compared to 990 nM for the parent hit **16**.

In summary, from the 15 selected series, ten confirmed their activity within the hit criteria by showing variability in pEC_50_ ± 0.5 or by displaying a higher potency against the *P. falciparum* NF54 with LEI for each validated hit between 14.0 and 26.3.

The compound initially identified as the most potent from HTS turned out to be inactive and possibly a false positive. One chemical entity (**1**) was unstable and dimerized on standing to produce a more active compound. Two series showed weak activity and therefore did not meet the potency criteria as a hit. One compound (**4a**) was synthesized as a racemate with defined relative stereochemistry, which did not confirm activity, however, activity related to the untested *cis*-diasteromers cannot be excluded.

Furthermore, applying informed structural modifications to a hit that would not otherwise pass strict cut-off filters (e.g., Ro5, BFGs) has proven to be successful in generating new validated hits with improved properties.

To determine if the 15 validated hits were truly novel, a search for exact matches in the recently launched malaria data portal developed and maintained by ChEMBL was performed [32]. Using the automated KNIME workflow mentioned above, three exact matches were found in public domain data (see Table 2). **12** was initially included into the MMV Open Access Malaria Box [33,34]. **14** had no stereochemistry assigned. Furthermore, seven hits had at least one nearest neighbour within a 0.85 Tanimoto similarity threshold using the standard MDL Symyx database fingerprints provided by the ChEMBL interface.

## Conclusion

A selection cascade has been applied to prioritize hits resulting from the screening of a diverse chemical library against blood-stage *P. falciparum*. Emphasis has been placed on chemical novelty and therefore computational clustering, the mining of known anti-malarial chemotypes and the application of relaxed physicochemical filters were key to the process. Ultimately, synthesis of near analogues solved chemical or biological liabilities that would have prevented them being in the final set of hits. The hits will be fully profiled in additional *Plasmodium* assays as well as in in vitro distribution metabolism pharmacokinetic (DMPK) assays. In particular, MMV is prosecuting most of the series and encourages research groups to contact them should they be interested in these hits. The data are available in the ChEMBL-NTD website [35].

## Competing interests

The authors declare that they have no competing interests.

## Authors’ contributions

VA, SD carried out and analysed the HTS screening. SS carried out the confirmatory assay. GP performed the cheminformatic analysis. SB, SP and YS synthesized the compounds. JNB, TS, DW and PW participated in the design of the study and performed the triaging. TS, and VA drafted and edited the manuscript. All authors read and approved the final manuscript.

## Supplementary Material

Additional file 1 **Experimental procedures and characterization data for the compounds in Figure** 8**and Table** 2**.** Experimental procedures for *Plasmodium falciparum* cultivation and growth assay. Click here for file
