# Supplementary material for: Screening and hit evaluation of a chemical library against blood-stage Plasmodium falciparum
Source: Malar J. 2014 May 27;13:190. doi: 10.1186/1475-2875-13-190 (PMC4094919; doi:10.1186/1475-2875-13-190)
Supplement: Additional file 1 — Experimental procedures and characterization data for the compounds in Figure 8and Table 2. Experimental procedures for Plasmodium falciparum cultivation and growth assay. [file 1475-2875-13-190-S1.docx]

**Additional file 1**

**Screening and hit evaluation of a chemical library against blood-stage *Plasmodium falciparum***

Vicky M Avery,^1^ Sridevi Bashyam,^2^ Jeremy N Burrows,^4^ Sandra Duffy,^1^ George Papadatos,^3^ Shyni Puthukkuti,^2^ Yuvaraj Sambandan,^2^ Shivendra Singh,^2^ Thomas Spangenberg^4^*, David Waterson^4^ and Paul Willis^4^

**^1^ Discovery Biology, Eskitis Institute for Drug Discovery, Griffith University, Nathan, Queensland 4111, Australia**

^2^ Syngene International Ltd, Plot No.2 & 3, Bommasandra IV Phase, Jigani Link Road, Bangalore, 560 100 - India

**^3^** Chemogenomics group, European Bioinformatics Institute (EMBL-EBI**),** European Molecular Biology Laboratory, Wellcome Trust Genome Campus, Hinxton, Cambs. CB10 1SD - United Kingdom

^4^ Me**dicines for Malaria Venture | MMV**, ICC - Block G, 3rd Floor, route de Pré-Bois 20 - PO Box 1826, 1215 Geneva 15 – Switzerland

*corresponding author: spangenbergt@mmv.org

**Table of content**

**Experimental Procedures and Characterization data for the compounds in Figure 8 and Table 2**

**Material and Methods**

- 2-((3-(hydroxymethyl)-2-methyl-1*H*-indol-1-yl)methyl)benzonitrile **(1)**
- 2,2'-((3,3'-methylenebis(2-methyl-1*H*-indole-3,1-diyl))bis(methylene))dibenzonitrile **(2)**
- *N*-(4-((*N*-(3-amino-2,2-dimethylpropyl)-3,3-dimethylbutanamido)methyl)phenyl)-4 cyanobenzamide **(3)**
- Rac (2*R*,3*R*)-1-acetyl-3-phenylindoline-2-carboxylic acid **(4a)**
- *N*-(2,4-dimethoxyphenyl)-1,5-diphenyl-1*H*-pyrazole-4-carboxamide **(5)**
- 1-(4-((2-benzyloxazol-4-yl)methyl)piperazin-1-yl)ethanone **(6)**
- *N*-(5-oxo-2-phenyl-4,5-dihydro-[1,2,4]triazolo[1,5-a]pyrimidin-7-yl)cyclohexanecarboxamide **(7)**
- *N*-1-(5-(6-methoxypyridin-3-yl)imidazo[2,1-b][1,3,4]thiadiazol-2-yl)cyclohexane-1,4-diamine **(8)**
- 2-ethoxy-*N*-(2-(3-methoxyphenoxy)-5-(trifluoromethyl)phenyl)nicotinamide **(9)**
- *N*-((3-(3-(1,4-diazepane-1-carbonyl)phenyl)-1,2,4-oxadiazol-5-yl)methyl)-*N*-(2-(piperidin-1-yl)ethyl)benzamide **(10)**
- *N*-(2-(azepan-1-yl)-2-(thiophen-3-yl)ethyl)-4-(trifluoromethyl)benzamide **(11)**
- 5'-methoxy-5*H*-spiro[benzo[4,5]imidazo[1,2-c]quinazoline-6,3'-indolin]-2'-one **(12)**
- 4-((4-methylthiazol-2-yl)thio)thieno[2,3-d]pyrimidine **(13)**
- (1*S*,3*S*)-1-(2,4-dichlorophenyl)-2,3,4,9-tetrahydro-1*H*-pyrido[3,4-b]indole-3-carboxylic acid **(14a)** and (1*R*,3*S*)-1-(2,4-dichlorophenyl)-2,3,4,9-tetrahydro-1*H*-pyrido[3,4-b]indole-3-carboxylic acid **(14b)**
- *N*-cyclopropyl-4-((2,5-dimethyl-4-tosylpiperazin-1-yl)methyl)benzamide **(15)**
- *N*-cyclopropyl-4-((4-tosylpiperazin-1-yl)methyl)benzamide **(15c)**
- 1'-benzyl-N-(3-chlorophenyl)spiro[indoline-3,4'-piperidine]-1-carboxamide **(17)**

**Parasitology**

- Cultivation of *Plasmodium falciparum*
- *Plasmodium falciparum* growth assay

**Material and Methods**

All reactions were carried out under nitrogen atmosphere or in sealed vials unless noted otherwise. Dry solvents and reagents were of commercial quality and were used as purchased. Reactions were magnetically stirred and monitored by thin-layer chromatography (TLC) using Merck silica gel 60 F254 by fluorescence quenching under UV light or by LCMS detection, except if indicated otherwise. LCMS-analyses were run on Agilent 1100/1200 series. In addition, TLC plates were stained using phosphomolybdic acid or potassium permanganate stain. Chromatographic purification of products (flash chromatography) was performed on Biotage Isolera systems and ethyl acetate/heptanes gradients. Concentration under reduced pressure was performed by rotary evaporation at 40°C at the appropriate pressure unless otherwise stated. The purity of the compounds reported in the manuscript was established through HPLC-MS methodology. HPLC-analyses were run according to the LCMS method. ^1^H-NMR (in DMSO-D_6_) and mass spectra are in agreement with the structures and were recorded on a Bruker AMX 400 MHz NMR spectrometer (TMS as an internal standard). All the compounds reported in the manuscript have a purity ≥95% unless noted otherwise.

**2-((3-(hydroxymethyl)-2-methyl-1H-indol-1-yl)methyl)benzonitrile (1)**

**(i)** To a solution of 2-methyl indole (1.0 g, 7.63 mmol) in DMF (50 mL) was added phosphorous oxychloride (1.06 mL, 11.45 mmol) drop wise after cooling to 0^°^C and stirred at room temperature for 5 hrs. After completion of the reaction, the excess phosphorus oxychloride was removed under high vacuum and the residue was diluted with ice-cold water and basified with NaHCO_3sat_ solution. The aqueous layer was extracted with dichloromethane, the combined organic layer was washed with water and followed by brine solution, and was dried over anhydrous Na_2_SO_4_, and the solvent was removed under reduced pressure to afford 2-methyl-1H-indole-3-carbaldehyde (1.0 g, 83%). **(ii)** To a suspension of sodium hydride (0.08 g, 1.9 mmol) in DMSO (10 mL) was added the 2-methyl-1H-indole-3-carbaldehyde (0.3 g, 1.8 mmol) after cooling to 0^°^C. After stirring the reaction mixture for *ca* 10 min, 2-(bromomethyl)benzonitrile (0.442 g, 2.2 mmol) in DMSO (5 mL) was added drop wise and stirred at room temperature for 2 hrs. The reaction was then quenched with saturated NH_4_Cl solution, diluted with ethyl acetate, washed with water, brine and dried over anhydrous Na_2_SO_4_. The combined organic layer was dried on Na_2_SO_4_ and the crude material purified by column chromatography to afford the desired adduct as a pale yellow solid (0.38 g, 74%). **(iii)** To a solution of 2-((3-formyl-2-methyl-1H-indol-1-yl)methyl)benzonitrile (0.05 g, 0.1 mmol) in isopropanol (5 mL) at 0 **̊**C, NaBH_4_ (0.008 g, 0.2 mmol) was added portion wise and stirred for 1 hr. The solvent was removed under *vacuo* and diluted the residue with ethyl acetate. The organic layer was then washed with water and the combined organic layer was dried on Na_2_SO_4._ The solvent was removed under reduced pressure to obtain the crude material which was purified by column chromatography to afford **1** (0.05 g, 49.7%). ^1^H NMR (400 MHz, DMSO-*d*6) δ ppm: 7.91 (d, *J* = 7.60 Hz, 1H), 7.60-7.62 (m, 1H), 7.52 (t, *J* = 8.44 Hz, 1H), 7.44 (t, *J* = 7.60 Hz, 1H), 7.30-7.32 (m, 1H), 7.02-7.04 (m, 1H), 6.39 (d, *J* = 7.80 Hz, 1H), 5.61 (s, 2H), 4.67 (s, 3H), 2.31 (s, 3H).

**2,2'-((3,3'-methylenebis(2-methyl-1H-indole-3,1-diyl))bis(methylene))dibenzonitrile (2)**

**(i)** To a solution of 2-methyl indole (1.0 g, 7.63 mmol) in DMF (50 mL) was added phosphorous oxychloride (1.06 mL, 11.45 mmol) drop wise after cooling to 0^°^C and stirred at room temperature for 5 hrs. The reaction mixture was then concentrated to remove phosphorous oxychloride, quenched with ice and extracted over dichloromethane. The combined organic layers were washed with saturated NaHCO_3_ solution followed by water and brine solution, dried over anhydrous Na_2_SO_4_, and concentrated in *vacuo* to afford 2-methyl-1H-indole-3-carbaldehyde (1.0 g, 83%). **(ii)** To the suspension of sodium hydride (0.08 g, 1.9 mmol) in DMSO (10 mL) was added 2-methyl-1H-indole-3-carbaldehyde (0.3 g, 1.8 mmol) after cooling to 0^°^C. After stirring the reaction mixture for *ca* 10 min, 2-(bromomethyl)benzonitrile (0.44 g, 2.2 mmol) in DMSO (5 mL) was added drop-wise and was stirred at room temperature for 2 hrs. The reaction mixture was then quenched with ice and diluted with ethyl acetate and washed with water. The combined organic layers were dried over Na_2_SO_4_ and the solvent evaporated under reduced pressure. The crude material was then purified by column chromatography to afford the desired adduct as a pale yellow solid (0.38 g, 74%). **(iii)** To a solution of 2-((3-formyl-2-methyl-1H-indol-1-yl)methyl)benzonitrile (0.1 g, 0.4 mmol) in methanol (5 mL) and THF (1 mL) at 0°C sodium borohydride (0.02 g, 0.5 mmol) was added portion wise and was stirred for 1 hr. Then the solvent was removed under *vacuo* and the reaction mixture was diluted with ethyl acetate and washed with water. The combined organic layer was dried over Na_2_SO_4_ and the solvent concentrated in *vacuo*. The crude material was purified by column chromatography to afford **2** (0.03 g, 16%). ^1^H NMR (400 MHz, DMSO-*d*6) δ ppm: 7.89-7.91 (m, 1H), 7.41-7.45 (m, 2H), 7.27-7.30 (m, 2H), 6.94-6.98, (m, 1H), 6.86-6.89 (m, 1H), 6.22-6.24 (m, 1H), 5.62 (s, 2H), 4.15 (s, 1H), 2.36 (s, 3H). LC-MS APCI: Calculated for LC-MS APCI: Calculated for C35H28N4 504.23; Observed *m/z* [M-H]^+^ 503. Purity by LC-MS: 92.51%. Purity by HPLC: 98.45%.

***N*-(4-((*N*-(3-amino-2,2-dimethylpropyl)-3,3-dimethylbutanamido)methyl)phenyl)-4-cyanobenzamide (3)**

**(i)** To a solution of *p*-toluidine (3.0 g, 27.9 mmol) and 4-cyano benzoic acid (4.12 g, 27.9 mmol) in dichloromethane (50 mL), was added triethylamine (11.6 g, 83.7 mmol), EDCI.HCl (10.7 g, 55.9 mmol) and HOBt (1.13 g, 8.3 mmol) and stirred at room temperature for 14 hrs. The reaction mixture was diluted with dichloromethane, washed with saturated NaHCO_3_ solution followed by 1.5N HCl water and brine solution. The combined organic layer was dried over anhydrous Na_2_SO_4_ and the solvent removed under reduced pressure to afford the desired adduct (4.0 g, 60%). **(ii)** To a solution of 4-cyano-*N*-(p-tolyl)benzamide (2.5 g, 10.5 mmol) in carbon tetrachloride (50 mL) was added NBS (2.074 g, 11.6 mmol), benzoyl peroxide (0.76 g, 3.15 mmol) and heated at reflux for 14 hrs. The reaction mixture was then concentrated to remove carbon tetrachloride and the residue was diluted with ethyl acetate, washed with water and the organic layer dried over anhydrous Na_2_SO_4_. The solvent was removed under reduced pressure to obtain the crude material. The crude material was then purified by column chromatography to afford *N*-(4-(bromomethyl)phenyl)-4-cyanobenzamide (1.1 g, 33%). **(iii)** To a solution of the latter compound (0.27 g, 8.57 mmol) in acetonitrile (5 mL), K_2_CO_3_ (0.177 g, 12.8 mmol), *tert*-butyl (3-amino-2,2-dimethylpropyl)carbamate acid (0.190 g, 9.42 mmol) was added and refluxed for 14 hrs. The reaction mixture was then concentrated to remove acetonitrile, diluted with ethyl acetate, washed with water and the organic layer dried over anhydrous Na_2_SO_4_. The solvent was removed under reduced pressure to obtain the crude product. The crude material obtained was then purified by column chromatography to afford *tert*-butyl (3-((4-(4-cyanobenzamido)benzyl)amino)-2,2-dimethylpropyl)carbamate (0.290 g, 77.5%). **(iv)** To a solution of the previously obtained compound (0.29 g, 0.66 mmol) and *tert*-butyl acetic acid (0.11 mL, 0.86 mmol) in dichloromethane (5 mL), triethylamine (0.276 mL, 1.99 mmol), EDCI.HCl (0.25 g, 1.32 mmol) and HOBt (0.026 g, 0.19 mmol) were added and stirred at room temperature for 14 hrs. The reaction mixture was diluted with dichloromethane, washed with saturated NaHCO_3_ solution followed by water and brine solution. The crude material obtained was purified by column chromatography to afford tert-butyl (3-(*N*-(4-(4-cyanobenzamido)benzyl)-3,3-dimethylbutanamido)-2,2-dimethylpropyl)carbamate (0.270 g, 76.2%). **(v)** To a solution of Boc protected **3** (0.27 g, 0.50 mmol) in dichloromethane (2 mL), trifluoroacetic acid (0.077mL, 1 mmol) was added after cooling the reaction mixture to 0°C and stirred at room temperature for 2 hrs. The reaction mixture was then diluted with dichloromethane, washed with 10% NaHCO_3_ solution followed by water and brine solution. The combined organic layer was dried on anhydrous Na_2_SO_4_ and solvent removed by reduced pressure to afford **3** (0.2 g, 91%). ^1^H NMR (400 MHz, DMSO-*d*6) δ ppm: 10.24 (s, 1H), 8.10 (d, *J* = 8.2 Hz, 2H), 7.96 (d, *J* = 8.24 Hz, 2H), 7.71-7.73 (m, 2H), 7.15-7.17 (m, 2H), 4.64 (s, 2H), 3.26 (s, 3H), 2.38 (s, 2H), 2.22 (s, 2H), 1.90 (s, 2H), 1.01 (s, 9H), 0.88 (s, 6H). LC-MS APCI: Calculated for C26H34N4O2 434.57; Observed *m/z* [M+H]^+^ 435.4. Purity by LC-MS: 99.33%. Purity by HPLC: 99.70%.

**Rac** **(2*R*,3*R*)-1-acetyl-3-phenylindoline-2-carboxylic acid (4a)**

**(i)** To a solution of 2-amino benzophenone (5.0 g, 25.3 mmol) in dry DMF (50 mL) was added drop-wise ethyl bromoacetate (2.83 mL, 25.3 mmol). The reaction mixture was heated to 80°C for 18 hrs. The solvent was evaporated, and the residue was taken up in ethyl acetate, and washed with water and brine. The combined organic layers were dried over Na_2_SO_4_ and the crude material was purified by column chromatography to afford ethyl 2-((2-benzoylphenyl)amino)acetate (6.0 g, 84%) as a yellow oil. **(ii)** To a solution of ethyl 2-((2-benzoylphenyl)amino)acetate (6.0 g, 21.1 mmol) in acetic anhydride (80 mL), 2 drops of conc. H_2_SO_4_ were added and the mixture was heated to 100 °C for 3 hrs. The solvent was evaporated, and the residue was extracted using ethyl acetate, and washed with water and brine. The combined organic layers were dried on Na_2_SO_4_ and the crude material purified by column chromatography to give ethyl 2-(*N*-(2-benzoylphenyl)acetamido)acetate (5.0 g, 72.5%) as brown oil. **(iii)** To a solution of the latter compound (5.0 g, 15.3 mmol) in DMF (25 mL), K_2_CO_3_ (5.3 g, 38.4 mmol) was added and the reaction mixture heated to 50° C for 3 hrs. Then it was poured in ice water and the pH was adjusted to 1 using 1.5N HCl. The resulting solid was filtered and the aqueous layer was extracted with ethyl acetate. The combined organic layer was dried over Na_2_SO_4_ and the solid obtained was triturated with Et_2_O which gave the desired adduct (3.6 g, 72%) as a white solid. **(iv)** To a solution of ethyl 1-acetyl-3-hydroxy-3-phenylindoline-2-carboxylate (1.0 g, 3.0 mmol) in Et_2_O (10 mL), HCl in Et_2_O (5 mL) was added drop wise at -5° C. Then the solvent was evaporated and the crude mixture was purified by column chromatography to give 1-(2-(ethoxymethyl)-3-phenyl-1H-indol-1-yl)ethanone hydrate (0.8 g, 84.7%) as white solid. **(v)** A mixture of the latter compound (0.8 g, 2.5 mmol) and 10% Pd/C (0.16 g) was stirred at room temperature in methanol (20 mL) and the mixture was hydrogenated under 4.13 bar pressure for 3 hrs. The catalyst was filtered through celite^®^ and washed with methanol. The filtrate and washings were combined and the solvent evaporated to give the desired product (0.38 g, 47%) as white solid. **(vi)** To a solution of ethyl 1-acetyl-3-phenylindoline-2-carboxylate (0.31 g, 1 mmol) in DME:water (20:5 mL), LiOH (0.328 g, 8.0 mmol) was added and stirred at room temperature for 2 hrs. Then DME was removed under reduced pressure and the resulting aqueous layer was acidified with citric acid and extracted with ethyl acetate. The combined organic layer was washed with brine and dried over Na_2_SO_4_. The organic layer was then concentrated under reduced pressure to afford **4a** (0.16 g, 56.9%) as orange solid. ^1^H NMR (400 MHz, DMSO-*d*6) δ ppm: 7.32 (t, *J* = 7.60 Hz, 2H), 7.23-7.27 (m, 2H), 7.18 (m, 2H), 7.09 (d, *J* = 7.32 Hz, 1H), 6.99-7.03 (m, 1H), 4.90 (d, *J* = 2.96 Hz, 1H), 4.63 (s, 1H), 2.19 (s, 3H). LC-MS APCI: Calculated for LC-MS APCI: Calculated for C17H15NO3 281; Observed *m/z* [M+H]^+^ 282. Purity by LC-MS: 96.34%. Purity by HPLC: 99.92%.

***N*-(2,4-dimethoxyphenyl)-1,5-diphenyl-1H-pyrazole-4-carboxamide (5)**

**(i)** To a solution of 1,5-diphenyl-1*H*-pyrazole-4-carboxylic acid (0.05 g, 0.1 mmol) in dichloromethane under nitrogen, a solution of 2,4-dimethoxy aniline (0.03 mL, 0.2 mmol), triethylamine (0.25 mL, 0.2 mmol) and 1-propanephosphonic anhydride (T_3_P) (0.035 g, 0.2 mmol) were added drop-wise and allowed to stir at room temperature for 14 hrs. The reaction mixture was diluted with dichloromethane, washed with saturated sodium bicarbonate solution followed by water and brine solution. The combined organic layer was dried over anhydrous Na_2_SO_4_ and the crude material was then purified by column chromatography to afford **5** as a solid (0.02 g, 26.3%). ^1^H NMR (400 MHz, DMSO-*d*6) δ ppm: 8.29 (s, 1H), 8.23 (s, 1H), 7.86 (d, *J* = 8.84 Hz, 1H), 7.32-7.48 (m, 8H), 7.23 (dd, *J* = 1.44, 7.80 Hz, 2H), 6.52-6.53 (m, 1H), 6.46 (dd, *J* = 2.64, 8.64 Hz, 1H), 3.71 (s, 3H), 3.57 (s, 3H). LC-MS APCI: Calculated for C24H21N3O3, 399.16; Observed *m/z* [M+H]^+^ 400.3. Purity by LC-MS: 98.07%. Purity by HPLC: 97.45%.

**1-(4-((2-benzyloxazol-4-yl)methyl)piperazin-1-yl)ethanone (6)**

**(i)** Phenyl acetamide (1.0 g, 7.39 mmol) was taken in 1,3-dichloroacetone (1.87 g, 14.7 mmol) and heated at 130°C in sealed tube for 14 hrs. Reaction mixture was diluted with dichloromethane, washed with water and brine solution. The combined organic layer was dried on anhydrous Na_2_SO_4_ and the solvent removed under reduced pressure. The crude material was purified by column chromatography to afford 2-benzyl-4-(chloromethyl)oxazole (0.1 g, 7%). **(ii)** To a solution of the latter compound (0.1 g, 0.48 mmol) in dioxane (2 mL) were added triethylamine (0.1 mL, 0.72 mmol) and *N*-acetyl piperazine (0.073 g, 0.57 mmol) and the reaction mixture heated at 80°C for 14 hrs. The reaction mixture was then concentrated to remove dioxane, diluted with dichloromethane, washed with water and brine solution. The combined organic layer was dried on anhydrous Na_2_SO_4_ solvent and removed under reduced pressure. The crude material was purified by column chromatography to afford the compound **6** (0.04 g, 27.7%). ^1^H NMR (400 MHz, DMSO-*d*6) δ ppm: 7.47 (s, 1H), 7.25-7.37 (m, 5H), 4.11 (s, 2H), 3.66 (s, 2H), 3.48 (s, 4H), 2.53-2.48 (m, 4H), 2.09 (s, 3H). LC-MS APCI: Calculated for C22H19F3N2O4 432.39; Observed *m/z* [M+H]^+^ 433.2. Purity by LC-MS: 98.23%. Purity by HPLC: 96.90%.

***N*-(5-oxo-2-phenyl-4,5-dihydro-[1,2,4]triazolo[1,5-a]pyrimidin-7-yl)cyclohexanecarboxamide (7)**

**(i)** To a suspension of benzohydrazide (1.0 g, 7.3 mmol) and methyl carbamiimidothioate (1.05 g, 7.3 mmol) in water (20 mL), NaOH (0.58 g, 14.6 mmol) was added and stirred at room temperature for 12 hrs. The white solid formed was filtered and dried to afford the desired adduct (0.6 g, 46%). **(ii)** 2-benzoylhydrazinecarboximidamide (0.6 g, 3.3 mmol) in water (5 mL) was taken in a microwave vial and heated to 200°C for 1 hr in microwave. The pale yellow solid formed was filtered and dried to afford 3-phenyl-1*H*-1,2,4-triazol-5-amine (0.5 g, 92.5%)**. (iii)** The latter compound (0.5 g, 3.1 mmol) was taken in ethyl cyanoacetate (12.5 mL, 25 vol) and heated at 190°C for 14 hrs. After the completion of the reaction, the solid formed was filtered, washed with Et_2_O to give 7-amino-2-phenyl-[1,2,4]triazolo[1,5-a]pyrimidin-5(4*H*)-one as yellow solid (0.55 g, 80%)**. (iv)** The mixture of the latter compound (0.25 g, 1.1 mmol) and cyclohexane carboxylic acid (12.5 mL, 25 vol) was taken in POCl_3_ (10 mL) and heated to 190°C for 14 hrs. After the completion of reaction, excess phosphorus oxychloride was removed under high vacuum and the residue was diluted with cold water (5 mL) and basified with NaHCO_3_ solution. The aqueous layer was extracted with ethyl acetate (25 mL), the combined organic layer was washed with water and followed by brine solution, and was dried over anhydrous Na_2_SO_4_, and the resulting solid was washed with Et_2_O to afford **7** (0.26 g, 70%) as yellow solid.^1^H NMR (400 MHz, DMSO-*d*6) δ ppm: δ 12.86 (s, 1H), 10.42 (s, 1H), 8.13 (d, *J* = 7.8 Hz 2H), 7.50-7.56 (m, 3H), 6.71 (s, 1H), 2.85-2.91 (m, 1H), 1.87 (d, *J* = 12.08 Hz, 2H), 1.76 (d, *J* = 12.04 Hz, 2H), 1.66 (d, *J* = 11.12 Hz, 1H), 1.18-1.43 (m, 5H). LC-MS APCI: Calculated for LC-MS APCI: Calculated for C18H19N5O2 337.15; Observed *m/z* [M+H]^+^ 338. Purity by LC-MS: 97.1%. Purity by HPLC: 98.46%.

***N*1-(5-(6-methoxypyridin-3-yl)imidazo[2,1-b][1,3,4]thiadiazol-2-yl)cyclohexane-1,4-diamine (8)**

**(i)** A mixture of 5-bromo-1,3,4-thiadiazol-2-amine (10.0 g, 55.5 mmol) and chloro-acetaldehyde (50% solution in water) (8.6 mL, 166 mmol) in water (100 mL) was heated to reflux for 24 hrs. The reaction mixture was diluted with dichloromethane, washed with saturated NaHCO_3_ solution followed by water and brine solution. The combined organic layer was dried on anhydrous Na_2_SO_4_ and the solvent removed under reduced pressure. The crude material was then purified by column chromatography to afford 2-bromoimidazo[2,1-b][1,3,4]thiadiazole (1.46 g, 12.8%). **(ii)** To a solution of the previous product (1.0 g, 4.9 mmol) in DMF (3.5 mL) was added NIS (1.24 g, 4.9 mmol) and stirred at room temperature for 6 hrs. The reaction mixture was diluted with ethyl acetate, washed with 10% sodium thiosulphate solution followed by water and brine solution. The combined organic layer was dried on anhydrous Na_2_SO_4_ and the solvent removed under reduced pressure to yield 2-bromo-5-iodoimidazo[2,1-b][1,3,4]thiadiazole (1.49 g, 92.5%). **(iii)** A mixture of the latter compound (1.0 g, 3.03 mmol) in acetonitrile (20 mL), triethylamine (1.26 mL, 9.09 mmol) and 1-Boc-1,4-cyclohexanediamine (0.780 g, 3.64 mmol) was refluxed at 100^°^C for 14 hrs. The reaction mixture was cooled to room temperature and the solvent removed under reduced pressure. The residue obtained was dissolved in dichloromethane, washed with water and the organic layer was dried over anhydrous Na_2_SO_4_. The crude material thus obtained was purified by column chromatography to afford *tert*-butyl (4-((5-iodoimidazo[2,1-b][1,3,4]thiadiazol-2-yl)amino)cyclohexyl)carbamate (0.75 g, 53.1%). **(iv)** To a 50 ml round bottom flask was added the previously obtained compound (0.40 g, 0.86 mmol), 6-methoxy pyridine-3-boronic acid pinacol ester (0.22 g, 0.94 mmol) and potassium carbonate (0.031 g, 0.22 mmol) were added and this mixture was suspended in dioxane (8 mL) and water (4 mL). After degassing the reaction mixture for 15 min, Pd(dppf)Cl_2_ (0.070 g, 0.086 mmol) was added and stirred at 110^°^C for 14 hrs. The reaction was cooled to room temperature diluted with water and ethyl acetate and extracted with ethyl acetate. The combined organic layer was washed with water and brine solution, then dried over anhydrous Na_2_SO_4_ and evaporated. The residue was purified by column chromatography to get *tert*-butyl (4-((5-(6-methoxypyridin-3-yl)imidazo[2,1-b][1,3,4]thiadiazol-2-yl)amino)cyclohexyl)carbamate (0.3 g, 78.5%). **(v)** To a solution of the latter compound (0.13 g, 0.29 mmol) in dioxane (2 mL), HCl in dioxane (2 mL) was added after cooling to 0^°^C and stirred for 2 hrs. The reaction mixture was concentrated to remove dioxane yielding **8** (0.13 g, 100%) as a brown solid. ^1^H NMR (400 MHz, DMSO-*d*6) δ ppm 9.18 (d, *J* = 8.00 Hz, 1H), 8.83-8.84 (m, 1H), 8.21-8.25 (m, 4H), 8.03 (s, 1H), 7.00 (d, *J* = 8.7 Hz, 1H), 3.88 (s, 3H), 3.53 (s, 1H), 3.01-3.02 (m, 1H), 2.14-2.17 (m, 2H), 2.02-2.05 (m, 2H), 1.33-1.51 (m, 4H). LC-MS APCI: Calculated for C16H20N6OS 344.43; Observed *m/z* [M+H]^+^ 445.2. Purity by LC-MS: 95.05%.

**2-ethoxy-*N*-(2-(3-methoxyphenoxy)-5-(trifluoromethyl)phenyl)nicotinamide (9)**

**(i)** To a solution of 3-methoxyphenol (0.2 g, 1.61 mmol) and 1-chloro-2-nitro-4(trifluoromethyl) benzene in dioxane (4 mL), K_2_CO_3_ (0.33 g, 2.41 mmol) was added at 0°C and refluxed for 12 hrs. The solvent was removed and evaporated under reduced pressure and the residue extracted with ethyl acetate and washed with water followed by brine solution. The crude material was purified by column chromatography to afford 1-(3-methoxyphenoxy)-4-methyl-2-nitrobenzene (0.32 g, 63.49%) as a brown-yellowish liquid. **(ii)** A mixture of 1-(3-methoxyphenoxy)-4-methyl-2-nitrobenzene (0.3 g, 0.96 mmol), iron powder (0.32 g, 0.96 mmol), ammonium chloride (0.051 g, 0.96 mmol) and EtOH/H_2_O solution (3 mL, 10:1) was stirred at 80°C for 3 hrs. After completion of reaction, iron powder was filtered and the residue extracted with ethyl acetate, dried over anhydrous Na_2_SO_4_ and concentrated under reduced pressure to afford 2-(3-methoxyphenoxy)-5-(trifluoromethyl)aniline (0.25 g, 92.71%) as yellow liquid. **(iii)** To a solution of the latter adduct (0.2 g, 0.71 mmol) in dichloromethane (2 mL) was added propyl phosphonic anhydride (T3P) (0.56 g, 1.77 mmol), 2-ethoxy-nicotinic acid (0.142 g, 0.85 mmol) and triethylamine (0.11 g, 1.06 mmol) at 0°C and stirred for 5 hrs. The mixture was extracted with dichloromethane (2 x 5 mL), dried over anhydrous Na_2_SO_4_ and concentrated under reduced pressure. The remaining residue was subjected to column chromatography to furnish to afford **9** (0.1 g, 32.78%) as a yellow solid.^1^H NMR (400 MHz, DMSO-*d*6) δ ppm: 8.96 (s, 1H), 8.49 (d, *J* = 7.6 Hz, 1H), 8.40-8.42 (m, 1H), 7.38-7.45 (m, 2H), 7.24-7.27 (m, 1H), 6.93-6.95 (m, 1H), 6.85-6.90 (m, 2H), 6.75-6.80 (m, 1H), 4.46 (q, *J* = 7.03 Hz), 3.77 (s, 3H), 1.22 (t, *J* = 7.05 Hz, 3H). LC-MS APCI: Calculated for C22H19F3N2O4 432.39; Observed *m/z* [M+H]^+^ 433.2. Purity by LC-MS: 98.23%. Purity by HPLC: 96.90%.

***N*-((3-(3-(1,4-diazepane-1-carbonyl)phenyl)-1,2,4-oxadiazol-5-yl)methyl)-*N*-(2-(piperidin-1-yl)ethyl)benzamide (10)**

**(i)** To a solution of 3-cyano benzoic acid (4.0 g, 27 mmol) and *N*-boc diazepene (5.9 g, 29.9 mmol) in dichloromethane (50 mL), triethylamine (11.3 mL, 81.5 mmol) was added and stirred for 5 hrs. To this solution was added EDCI.HCl (6.7 g, 35.1 mmol) and HOBt (0.73 g, 5.4 mmol) and stirred at room temperature 14 hrs. The reaction mixture was diluted with dichloromethane, and washed with saturated NaHCO_3_ solution followed by water and brine solution. The combined organic layer was dried on anhydrous Na_2_SO_4_ solvent and removed by reduced pressure. The crude material was purified by column chromatography to afford *tert*-butyl 4-(3-cyanobenzoyl)-1,4-diazepane-1-carboxylate (4.5 g, 50%). **(ii)** To a solution of the latter adduct in EtOH:H_2_O (30:10 mL) mixture was added hydroxyl amine hydrochloride (0.95 g,13.6 mmol) and triethylamine (3.16 mL, 22.7 mmol) and heated at 100^°^C for 3 hrs. After checking the conversion of the starting material, the reaction mixture was cooled to room temperature and concentrated under reduced pressure to yield (*Z*)-*tert*-butyl 4-(3-(N'-hydroxycarbamimidoyl)benzoyl)-1,4-diazepane-1-carboxylate (5.0 g) as crude white solid. **(iii)** To a solution of adduct from the previous step (3.0 g, 8.28 mmol) in ethanol (15 mL), triethylamine (1.72 mL, 12.4 mmol) was added and stirred at 0°C for 5 min. Then chloro acetylchoride was added to the reaction mixture at 0°C and heated at 80°C in a sealed tube for 3 hrs. The reaction mixture was cooled to room temperature, and the solvent removed under reduced pressure. The residue obtained was dissolved in dichloromethane, washed with water and the organic layer dried over anhydrous Na_2_SO_4_. The crude material obtained was purified by column chromatography to afford the desired *tert*-butyl 4-(3-(5-(chloromethyl)-1,2,4-oxadiazol-3-yl)benzoyl)-1,4-diazepane-1-carboxylate (0.83 g, 21% (combined yield step ii&iii)) as gummy liquid. **(iv)** To a solution of the latter compound (0.83 g, 1.99 mmol) in dichloromethane, was added triethylamine (1.2 mL, 5.9 mmol) and stirred for 5 min. To this stirred solution was added 2-(piperidin-1-yl)ethanamine (0.38 g, 2.96 mmol) drop-wise and stirred at room temperature for 14 hrs. After completion of starting material, the reaction mixture was diluted with dichloromethane and washed successively with water followed by brine solution. The combined organic layer was dried over anhydrous Na_2_SO_4_ and the solvent removed under reduced pressure to get the crude oil. The crude material was purified by column chromatography to afford *tert*-butyl-4-(3-(5-(((2-(piperidin-1-yl)ethyl)amino)methyl)-1,2,4-oxadiazol-3-yl)benzoyl)-1,4-diazepane-1-carboxylate (0.8 g, 79%) as brown liquid. **(v)** To a solution of the latter product (0.25 g, 0.48 mmol) in dichloromethane (10 mL), triethylamine (0.2 mL, 1.46 mmol) was added and allowed to stir for 5 min. The reaction mixture was cooled to 0^°^C followed by the addition of benzoyl chloride (0.06 g, 0.7 mmol) and the reaction mixture warmed to room temperature and stirred for 30 min. The reaction mixture was diluted with dichloromethane and washed with water and brine solution. The organic layer was collected, dried over anhydrous Na_2_SO_4_ and the solvent removed under reduced pressure. The crude material was purified by column chromatography to afford tert-butyl 4-(3-(5-(((1-phenylvinyl)(2-(piperidin-1-yl)ethyl)amino)methyl)-1,2,4-oxadiazol-3-yl)benzoyl)-1,4-diazepane-1-carboxylate (0.1 g, 40%) as brown liquid. **(vi)** To a solution of the latter adduct (0.1 g, 0.16 mmol) in dichloromethane (3 mL), trifluoroacetic acid (0.3 g, 2.4 mmol) was added at 0°C and stirred at room temperature for 2 hrs. After completion of the reaction, the solvent was removed under reduced pressure. The residue obtained was dissolved in dichloromethane and to this ion exchange resin was added and stirred for 30 min. The resin was then filtered off and the filtrate concentrated to give **10** (0.05 g, 60%) as a gummy liquid. ^1^H NMR (400 MHz, DMSO-*d*6) δ ppm: 8.07-8.05 (m, 1H), 7.97 (s, 1H), 7.66-7.58 (m, 2H), 7.48-7.44 (m, 5H), 5.02 (s, 2H), 3.67-3.46 (m, 4H), 2.84 (m, 4H), 2.32-2.24 (m, 4H), 1.73-1.71 (m, 2H), 1.43-1.39 (m, 4H), 1.33-1.27 (m, 4H). LC-MS APCI: Calculated for C29H36N6O3 516.65; Observed *m/z* [M+H]^+^ 517.4. Purity by LC-MS: 97.58%. Purity by HPLC: 99.79%.

***N*-(2-(azepan-1-yl)-2-(thiophen-3-yl)ethyl)-4-(trifluoromethyl)benzamide (11)**

**(i)** To a solution of 3-acetyl thiophene (2.0 g, 15.8 mmol) in dichloromethane:methanol mixture (200:80 mL), *tert*-butyl ammonium tribromide (8.4 g, 17.38 mmol) was added and stirred at room temperature for 3 hrs. After the completion of the reaction, the solvent was evaporated, washed with water, brine and dried over anhydrous Na_2_SO_4_. Concentration of the organic layer followed by trituration with diethyl ether gave 2-bromo-1-(thiophen-3-yl)ethanone (3.0 g, 92.5%) as a white solid. **(ii)** To a solution of 2-bromo-1-(thiophen-3-yl)ethanone (3.0 g, 15.1 mmol) in dichloromethane (20 mL), hexamethylenetetramine (2.1 g, 15.1 mmol) was added and stirred for 1 hr. The reaction mixture was filtered and washed with dichloromethane to yield a white solid which was then stirred in ethanol and HCl for 14 hrs. The resulting residue formed was filtered and the filtrate evaporated under reduced pressure to give 2-amino-1-(thiophen-3-yl)ethanone hydrochloride (1.9 g, 71%) as a yellow solid. **(iii)** The mixture of the latter HCl salt (1.0 g, 7.0 mmol), 4-trifluromethyl benzoic acid (1.33 g, 7.0 mmol), triethylamine (2.9 mL, 21.0 mmol) and 1-propanephosphonic anhydride (T_3_P) (2.2 mL) were taken in dichloromethane (15 mL) and allowed to stir at room temperature for 14 hrs. The reaction mixture was diluted with dichloromethane, washed with saturated sodium bicarbonate solution followed by water and brine solution. The combined organic layer was dried over anhydrous Na_2_SO_4_ and the crude material was then purified by column chromatography to afford *N*-(2-oxo-2-(thiophen-3-yl)ethyl)-4-(trifluoromethyl)benzamide as pale yellow solid (1.5 g, 68%). **(iv)** To a solution of the previous adduct (0.2 g, 0.64 mmol) in methanol (5 mL) under nitrogen atmosphere at 0^°^C, sodium borohydride (0.037 g, 0.96 mmol) was added portion wise and stirred for 1 hr. The solvent was evaporated, and the residue was extracted with ethyl acetate, and washed with water. The combined organic layer was dried with Na_2_SO_4_ and the crude material purified by column chromatography over to afford *N*-(2-hydroxy-2-(thiophen-3-yl)ethyl)-4-(trifluoromethyl)benzamide (0.19 g, 94.5%). **(v)** To a mixture of the previous adduct (0.59 g, 1.89 mmol), azepane (0.9 mL, 7.55 mmol) and triphenyl phosphine (2.02 g, 7.55 mmol) in THF (15 mL), was added diethyl azodicarboxylate (DEAD) (1.18 mL, 7.55 mmol) drop-wise at 0^°^C and stirred at room temperature for 16 hrs. The solvent was evaporated, and the residue was extracted in dichloromethane and washed with water. The combined organic layer was dried on Na_2_SO_4_ and the crude material was purified by preparative HPLC to afford **11** (0.016 g, 2%).^1^H NMR (400 MHz, DMSO-*d*6) δ ppm: 7.95 (s, 1H), 7.93 (s, 1H), 7.94 (d, *J*= 8.16 Hz, 2H), 7.82-7.83 (m, 1H), 7.79 (d, *J* = 8.2 Hz, 2H), 7.66-7.68 (m, 1H), 7.38 (dd, *J* = 1.32, 4.0 Hz, 1H), 4.32-4.35 (m, 1H), 3.88-3.93 (m, 1H), 3.05-3.75 (m, 2H), 3.13-3.16 (m, 1H), 1.79-1.91 (m, 4H), 1.66-1.75 (m, 5H), 1.37-1.40 (m, 2H). LC-MS APCI: Calculated for C20H23F3N2OS 396.15; Observed *m/z* [M+H]^+^ 397. Purity by LC-MS: 98.9%. Purity by HPLC: 91.12%.

**5'-methoxy-5*H*-spiro[benzo[4,5]imidazo[1,2-c]quinazoline-6,3'-indolin]-2'-one (12)**

**(i)** A mixture of anthranilic acid (0.3 g, 2.7 mmol), phenylene diamine (0.57 g, 4.1 mmol) and PPA (3.0 g, 10 vol) were taken in a microwave vial, mixed thoroughly with glass rod and was heated to 140°C for 2 hrs. After the completion of the reaction, it was basified with Na_2_CO_3_ solution, diluted with ethyl acetate, washed with water, brine and dried over anhydrous Na_2_SO_4_. Concentration of the organic layer gave a pale yellow oil which was purified by column chromatography to get 2-(1*H*-benzo[d]imidazol-2-yl)aniline as a pale yellow solid (0.28 g, 48.2%). **(ii)** To a solution of the previous adduct (0.1 g, 4.0 mmol) and 5-methoxy isatin (0.085 g, 4.0 mmol) in methanol (5 mL), acetic acid (0.5 mL) was added and heated to 60°C for 5 hrs. The solvent was removed under *vacuo* and the crude material was purified by column chromatography over to afford **12** (0.125 g, 71%) as pale yellow solid.^1^H NMR (400 MHz, DMSO-*d*6) δ ppm: 10.63 (s, 1H), 7.96 (d, *J* = 7.04 Hz, 1H), 7.80 (s, 1H), 7.66 (d, *J* = 7.96 Hz, 1H), 7.24-7.29 (m, 1H), 7.09-7.19 (m, 3H), 7.01-7.03 (m, 1H), 6.95-6.99 (m, 1H), 6.80-6.87 (m, 2H), 6.11 (d, 1H, *J* = 8.16 Hz), 3.68 (s, 3H). LC-MS APCI: Calculated for C22H16N4O2 368.13; Observed *m/z* [M+H]^+^ 368.39. Purity by LC-MS: 98.27%. Purity by HPLC: 98.68%.

**4-((4-methylthiazol-2-yl)thio)thieno[2,3-d]pyrimidine (13)**

**(i)** 2-Amino-thiophene-3-carboxylic acid methyl ester (0.5 g, 3.18 mmol) was added to formamide solution (10 mL) at 0°C. The reaction mixture was refluxed at 210°C for 6 hrs. The reaction mixture was cooled to room temperature, and removed the excess formamide under reduced pressure. The crude material was then purified by column chromatography to afford thieno[2,3-d]pyrimidin-4-ol (0.3 g, 62%) as yellow solid. **(ii)** thieno[2,3-d]pyrimidin-4-ol (0.5 g, 3.29 mmol) was taken in phosphorus oxychloride (5 mL) and heated to reflux for 4 hrs at 110°C. After the completion of reaction, excess phosphorus oxychloride was removed under high vacuum and the residue was diluted with cold water (5 mL) and basified with a NaHCO_3_ solution. The aqueous layer was extracted with ethyl acetate (25 mL), the combined organic layer was washed with water and followed by brine solution, and was dried over anhydrous Na_2_SO_4_. The crude residue was subjected to column chromatography to afford 4-chlorothieno[2,3-d]pyrimidine (0.29 g, 52%) as yellow solid. **(iii)** To a stirred solution of 4-chlorothieno[2,3-d]pyrimidine (0.075 g, 0.44 mmol) and 4-methyl-3*H*-thiazole-2-thione in ethanol (4 mL) was added sodium acetate (0.054 g, 6.59 mmol) at room temperature and refluxed at 80°C for 14 hrs. The reaction mixture was cooled to room temperature and concentrated under reduced pressure. The crude material was purified by column chromatography to afford compound **13** (0.070 g, 60%) as a white solid. ^1^H NMR (400 MHz, DMSO-*d*6) δ ppm: 8.90 (s, 1H), 8.08 (d, *J* = 8.00 Hz, 1H), 7.63 (s, 1H), 7.56 (d, *J* = 8.00 Hz, 1H), 2.46 (s, 3H). LC-MS APCI: Calculated for C10H7N3S3 265.38; Observed *m/z* [M+H]^+^ 266.0. Purity by LC-MS: 99.72%. Purity by HPLC: 99.71%.

**(1*S*,3*S*)-1-(2,4-dichlorophenyl)-2,3,4,9-tetrahydro-1*H*-pyrido[3,4-b]indole-3-carboxylic acid (14a) and (1*R*,3*S*)-1-(2,4-dichlorophenyl)-2,3,4,9-tetrahydro-1H-pyrido[3,4-b]indole-3-carboxylic acid (14b)**

**(i)** The mixture of L-tryptophan (1 g, 5.0 mmol), 2,4-dichlorobenzaldehyde (0.86 g, 5.0 mmol) and H_2_SO_4_ (0.5M, 5 mL) were taken in water (15 mL) and ethanol (10 mL) in a 100 mL round-bottom flask, and refluxed for 5 hrs. After adding concentrated ammonia (10 mL), the mixture was refluxed for another hour. The reaction mixture was concentrated and the resulting solution was extracted with ethyl ether. The volume of the aqueous phase was reduced to 10 mL and the adjusted to pH 5. The precipitate formed was then filtered, washed well with water and dried. **(ii)** The mixture of *cis* and *trans* isomers were separated by chiral preparative HPLC (CHIRAL PAK ADH® (250x 4.6)mm 5 micron, 80% of 0.1%TFA in Hexane and 20% of Ethanol, 1.0 ml/min). **14a/b** ^1^H NMR (400 MHz, DMSO-*d*6) δ ppm 10.67 (s, 1H), 10.41 (s, 1H), 7.67-7.69 (m, 2H), 7.47 (d, *J* = 8.00 Hz, 1H), 7.38 (d, *J* = 8.00 Hz, 2H), 7.29-7.31 (m, 2H), 7.01-7.03 (m, 4H), 6.77 (d, *J* = 8.00 Hz, 2H), 3.81-3.84 (m, 1H), 3.54-3.57 (m, 2H), 3.03-3.04 (m, 2H), 2.81-2.84 (m, 2H). LC-MS APCI: Calculated for C18H14Cl2N2O2 361.23; Observed *m/z* [M+H]^+^ 361.2. Purity by LC-MS: 97.5%. **14a** ^1^H NMR (400 MHz, DMSO-*d*6) δ ppm 7.73 (s, 1H), 7.55 (d, *J* = 8.00 Hz, 1H), 7.42 (dd, *J* = 2.00 Hz, 8.00 Hz, 1H), 7.26 (d, *J* = 8.08 Hz, 1H), 7.06-7.15 (m, 2H), 6.31 (s, 1H), 4.23-4.27 (m, 1H), 3.33 (dd, *J* = 1.60, 3.20 Hz, 1H), 3.15-3.17 (m, 1H), 2.64-2.68 (m,1H), 2.17-2.19 (m, 1H). LC-MS APCI: Calculated for C18H14Cl2N2O2 361.23; Observed *m/z* [M+H]^+^ 361.2. Purity by LC-MS: 99.21%. **14b** ^1^H NMR (400 MHz, DMSO-*d*6) δ ppm 7.72 (s, 1H), 7.57 (d, *J* = 7.76 Hz, 1H), 7.37 (dd, *J* = 2.12 Hz, 8.00 Hz, 1H), 7.28 (d, *J* = 8.04 Hz, 1H), 7.15-7.18 (m, 1H), 7.08-7.11 (m, 1H), 7.02 (d, *J* = 8.00 Hz, 1H), 6.37 (s, 1H), 3.93-3.97 (m, 1H), 3.45-3.50 (m, 1H), 3.21-3.27 (m, 1H), 2.64 (s, 1H), 2.19 (s, 1H). LC-MS APCI: Calculated for C18H14Cl2N2O2 361.23; Observed *m/z* [M+H]^+^ 361.2. Purity by LC-MS: 97.5%.

***N*-cyclopropyl-4-((2,5-dimethyl-4-tosylpiperazin-1-yl)methyl)benzamide (15)**

**(i)** To the solution of 2,5-dimethyl piperazine (1.0 g, 8.77 mmol) in acetonitrile (10 mL), triethylamine (2.4 mL, 17.54 mmol) and 4-bromo methyl benzoate (2.20 g, 9.64 mmol) was added and stirred at room temperature for 3 hrs. After the completion of the reaction, the solvent was evaporated, extracted with ethyl acetate, washed with water, brine and dried over anhydrous Na_2_SO_4_. Concentration of the organic layer gave methyl 4-((2,5-dimethylpiperazin-1-yl)methyl)benzoate (2.2 g, 59%) which was taken as such for next step. **(ii)** To a solution of the crude compound (2.2 g, 8.7 mmol) in dioxane (20 mL), was added tosyl chloride (2.0 g, 10.52 mmol) and stirred at 80^°^C for 14 hrs. After the completion of the reaction, the solvent was evaporated, extracted with ethyl acetate, washed with water, brine and dried over anhydrous Na_2_SO_4_. The crude product obtained was purified by column chromatography to afford methyl 4-((2,5-dimethyl-4-tosylpiperazin-1-yl)methyl)benzoate (0.4 g). **(iii)** To a solution of the latter compound (0.40 g, 0.96 mmol) in methanol:water (8 mL:4 mL), lithium hydroxide (0.16 g, 3.85 mmol) was added and stirred at room temperature for 14 hrs. The reaction mixture was concentrated and the residue obtained was dissolved in water, acidified with citric acid, extracted using ethyl acetate. The ethyl acetate layer was washed with water and dried over anhydrous Na_2_SO_4_. The solvent was removed under reduced pressure to afford 4-((2,5-dimethyl-4-tosylpiperazin-1-yl)methyl)benzoic acid (0.35 g, 90.43%). **(iv)** To a solution of the latter acid (0.35 g, 0.87 mmol) in dichloromethane was added triethylamine (0.36 mL, 2.60 mmol), cyclopropylamine (0.06 g, 1.04 mmol) and propylphosphonic anhydride (T_3_P) (0.83 mL, 1.30 mmol) and stirred at room temperature for 14 hrs. The reaction mixture was then washed with water and brine solution. The combined organic layer was dried over anhydrous Na_2_SO_4_ and the solvent was removed under reduced pressure to get the crude oil. The crude material was then purified by column chromatography to afford **15** (0.25 g, 65.27%). ^1^H NMR (400 MHz, DMSO-*d*6) δ ppm: 8.06 (s, 1H), 7.73 (d, *J* = 4.16 Hz, 2H), 7.65 (d, *J* = 8.00 Hz, 2H), 7.39 (d, *J* = 8.00 Hz, 2H), 7.34 (d, *J* = 8.12 Hz, 2H), 3.78-3.80 (m, 1H), 3.54 (m, 2H), 3.33 (m, 1H), 3.19 (m, 1H), 2.91 (t, *J* = 3.32 Hz, 1H), 2.85 (m, 1H), 2.67 (m, 1H), 2.50 (m, 3H), 2.13 (m, 1H), 1.03 (d, *J* = 6.6 Hz, 3H), 0.91 (d, *J* = 6.4 Hz 3H), 0.67 (q, *J* = 1.20 Hz, 2H), 0.56 (q, *J* = 4.00 Hz, 2H). LC-MS APCI: Calculated for C24H31N3O3S 441.59; Observed *m/z* [M+H]^+^ 442.2. Purity by LC-MS: 99.57%.

***N*-cyclopropyl-4-((4-tosylpiperazin-1-yl)methyl)benzamide (15c)**

**(i)** To the solution of *tert*-butyl piperazine-1-carboxylate (0.5 g, 2.68 mmol) in acetonitrile (5 mL), triethylamine (0.56 mL, 4.02 mmol) and 4-bromo methyl benzoate (0.675 g, 2.948 mmol) were added and the resulting solution was stirred at room temperature for 3 hrs. After the completion of the reaction, the reaction mixture was concentrated to remove acetonitrile. The residue was dissolved in ethyl acetate, washed with water and over brine solution. The organic layer was then dried over sodium sulphate and concentrated to yield the *tert*-butyl 4-(4-(methoxycarbonyl)benzyl)piperazine-1-carboxylate (0.80 g, 89%) which was taken as such for next step. **(ii)** To the solution of the latter adduct (0.80 g, 2.38 mmol) in methanol (8 mL) and water (4 mL), lithium hydroxide (0.40 g, 9.55 mmol) was added after cooling to 0°C. Then the reaction mixture was stirred at room temperature for 2 hrs. After the completion of the reaction, the reaction mixture was concentrated to remove methanol, added water, acidified with citric acid, extracted over ethyl acetate, water and brine. The organic layer was then separated, dried over sodium sulphate, concentrated to get the corresponding carboxylic acid (0.7 g, 91.6%). **(iii)** To the solution of 4-((4-(*tert*-butoxycarbonyl)piperazin-1-yl)methyl)benzoic acid (0.7 g, 2.18 mmol) in dichloromethane (10 mL), triethylamine (0.46 mL, 3.27 mmol), cyclopropyl amine (0.15 g, 2.61 mmol) were added followed by T3P (1.03 g, 3.27 mmol) after cooling the reaction mixture to 0°C. The reaction mixture was stirred at room temperature for 2 hrs. After the completion of the reaction, the reaction mixture was washed with water and brine solution. The organic layer was then separated, dried over sodium sulphate, concentrated and purified by column chromatography to afford the corresponding pure amide (0.7 g, 89.2%). **(iv)** To the solution of *tert*-butyl 4-(4-(cyclopropylcarbamoyl)benzyl)piperazine-1-carboxylate (0.7 g, 1.947 mmol) in dioxane (5 mL), HCl in dioxane (4 mL) was added drop wise after cooling to 0°C. Then the reaction mixture was stirred at room temperature for 4 hrs. After the completion of the reaction, the reaction mixture was concentrated to remove the dioxane to get the corresponding hydrochloride salt (0.58 g, 100%). **(v)** To the solution of *N*-cyclopropyl-4-(piperazin-1-ylmethyl)benzamide (0.15 g, 0.42 mmol) in dioxane (3 mL), triethylamine (0.17ml, 1.25 mmol) was added followed by tosyl chloride (0.09 g, 0.50 mmol) and heated at 80°C for 14 hrs. After the completion of the reaction, the reaction mixture was concentrated to remove dioxane and the residue was dissolved in ethyl acetate and washed with water, and brine solution. The organic layer was then separated, dried over sodium sulphate and the solvent removed to obtain the crude product. The crude product was purified by column chromatography to afford **15c** (0.12 g, 69.76%). ^1^H NMR (400 MHz, DMSO-d6) δ ppm: 8.36 (s, 1H), 7.71 (d, *J* = 8.00Hz, 2H), 7.60 (d, *J* = 8.00 Hz, 2H), 7.45 (d, *J* = 8.00 Hz, 2H), 7.27 (d, *J* = 8.00 Hz, 2H), 3.48 (s, 1H), 2.78-2.79 (m, 5H), 2.41-2.46 (m, 7H), 0.65-0.66 (m, 2H), 0.51-0.52 (m, 2H), LC-MS APCI: Calculated for C22H27N3O3S 413.54; Observed m/z [M+H]+ 414.2. Purity by LC-MS: 99.19%.

**1'-benzyl-N-(3-chlorophenyl)spiro[indoline-3,4'-piperidine]-1-carboxamide (17)**

**(i)** To the solution of spiro(indoline-3,4-piperidine) (0.25 g, 1.32 mmol) in dichloromethane (5 mL), was added triethylamine (0.20 g, 1.99 mmol), benzyl bromide (0.23 g, 1.32 mmol) at 0°C and stirred at room temperature for 3 hrs. Then the reaction mixture was diluted with dichloromethane, washed with water and brine solution. The combined organic layer was dried on anhydrous sodium sulphate and the solvent was removed under reduced pressure. The crude material was then purified by column chromatography to afford 1'-benzylspiro[indoline-3,4'-piperidine] (0.22 g, 61.11%) as yellowish solid. **(ii)** To a solution of the latter adduct (0.1 g, 0.36 mmol) in dichloromethane (2 mL) was added triethylamine (0.06 g, 0.61 mmol), 1-chloro-3-isocynatobenzene (0.06 g, 0.4 mmol) at 0°C and stirred at room temperature for 14 hrs. Then the reaction mixture was diluted with dichloromethane, washed with water and brine solution. The combined organic layer was dried on anhydrous sodium sulphate and the solvent was removed under reduced pressure. The crude material was then purified by column chromatography to afford **17** (0.04 g, 29.03%) as a yellowish solid. ^1^H NMR (400 MHz, DMSO-*d*6) δ ppm: 8.73 (s, 1H), 7.88 (d, *J* = 8.00 Hz, 1H), 7.75 (s, 1H), 7.55 (d, *J* = 5.60 Hz, 2H), 7.42 (d, *J* = 2.80 Hz, 1H), 7.28-7.30 (m, 2H), 7.25 (d, *J* = 7.20 Hz, 2H), 7.12-7.16 (m, 1H), 7.06 (d, *J* = 7.20 Hz, 1H), 6.92-6.96 (m, 1H), 3.99 (s, 2H), 3.53 (s, 2H), 2.83 (m, 2H), 2.10-2.15 (m, 2H), 1.83-1.89 (m, 2H), 1.59-1.62 (m, 2H). LC-MS APCI: Calculated for C26H26ClN3O 431.96; Observed *m/z* [M+H]^+^ 432.2. Purity by LC-MS: 94.60%. Purity by HPLC: 97.89%.

**Cultivation of *Plasmodium falciparum*:** *Plasmodium falciparum* strain NF54 was obtained from the Research and Reference Reagent Resource Center (MR4) (Manassas, VA, USA). The two strains were maintained *in vitro* by a modification of the method of Trager and Jensen. Cultures were maintained in A positive (A^+^) human erythrocytes suspended at 5% hematocrit in complete medium. Five g albumax II (Gibco-Invitrogen, Cat No# 11021037), 2.5mg gentamicin (Sigma Aldrich), 25 mM HEPES (Invitrogen), 5 mg hypoxanthine (Sigma), and 1 L of RPMI 1640 (Invitrogen, Cat No#11875085). Cultures were grown in 100mm petri-dishes (BD Falcon) at a volume of 15 ml and were kept in a standard gas environment of 4% CO_2_ and 3% O_2_ at temperature 37°C in a tri-gas incubator (Cat#3131, Thermo Scientific Forma Series II Water Jacketed). Parasite growth and morphology were observed daily using thin smears at 100X (oil immersion) magnification following staining with Giemsa stain.

***Plasmodium falciparum* growth assay:** The protocol assesses the compound efficacy against the growth of *P. falciparum in vitro*.

Parasite growth was detected in assay by the traditional [^3^H] hypoxanthine incorporation assay as previously described by Desjardins and colleagues (*Antimicrob. Agents Chemother.*, *16*(6), 710, 1979). To perform the [^3^H] hypoxanthine incorporation assay, the new anti-malarial agents were serially diluted 1:1 into hypoxanthine-free complete medium to a final volume of 100 μL (final anti-malarial agent concentration range, 10,000 nM to 4.8 nM may change in special case) in 96-well sterile cell culture plates. One-hundred μL of *P. falciparum* culture (0.3%p and 1.25%h-synchronized ring stage) is added per well, by addition, anti-malarial agents are diluted in such a way that the final DMSO concentration in the well does not exceed 0.1%. All cultures used in the study are albumax II adopted. The microtitre plates were incubated in chamber in a standard gas environment at 37°C for 72 hrs. After 48 hrs of incubation and prior to addition of 50 µL (0.5 µCi/well) [^3^H] -Hypoxanthine (specific activity, 20Ci/mmol,Conc.1.0 mCi/ml; American Radiolabeled Chemicals, Inc, St. Louis, MO, USA), culture growth was assessed by making the smears that ensures the culture has grown in % p and assay plate is further incubated for 24 hrs.

Following the incubation period, the plates were harvested with a FilterMate cell harvester (Perkin Elmer) onto unifilter-96 GF/B plates, washed with distilled water to remove excess radiochemical and plates were kept for drying 37°C overnight or 60°C for 1 hr. Fifty µL of Microscint scintillation cocktail (Microscint-High Efficiency LSC-Cocktail; Perkin Elmer) added in the unifilter-96 GF/B plates and kept for 15-20 min. The plates were counted in a Top Count NXT microplate scintillation and luminescence counter (Perkin Elmer). The mean values for [^3^H]-hypoxanthine incorporation in parasitized control and non-parasitized control erythrocytes were calculated.

Assay data were analysed using Graph pad prism ver.5 software. A variable sigmoid dose response curve is plotted keeping log concentrations at X-axis and % inhibition at Y-axis.
